# The impact of sample storage time on estimates of association in biomarker discovery studies

**DOI:** 10.1186/2043-9113-1-9

**Published:** 2011-03-08

**Authors:** Karl G Kugler, Werner O Hackl, Laurin AJ Mueller, Heidi Fiegl, Armin Graber, Ruth M Pfeiffer

**Affiliations:** 1 Institute for Bioinformatics and Translational Research, University for Health Sciences, Medical Informatics and Technology, EWZ 1, 6060, Hall in Tirol, Austria; 2 Department of Obstetrics and Gynecology, Innsbruck Medical University, Anichstrasse 35, 6020, Innsbruck, Austria; 3 Novartis Pharmaceuticals Corporation, Oncology Biomarkers and Imaging, One Health Plaza, East Hanover, NJ 07936, USA; 4 Biostatistics Branch, Division of Cancer Epidemiology and Genetics, National Cancer Institute, Bethesda, MD 20892, USA

## Abstract

**Background:**

Using serum, plasma or tumor tissue specimens from biobanks for biomarker discovery studies is attractive as samples are often readily available. However, storage over longer periods of time can alter concentrations of proteins in those specimens. We therefore assessed the bias in estimates of association from case-control studies conducted using banked specimens when maker levels changed over time for single markers and also for multiple correlated markers in simulations. Data from a small laboratory experiment using serum samples guided the choices of simulation parameters for various functions of changes of biomarkers over time.

**Results:**

In the laboratory experiment levels of two serum markers measured at sample collection and again in the same samples after approximately ten years in storage increased by 15%. For a 15% increase in marker levels over ten years, odds ratios (ORs) of association were significantly underestimated, with a relative bias of -10%, while for a 15% decrease in marker levels over time ORs were too high, with a relative bias of 20%.

**Conclusion:**

Biases in estimates of parameters of association need to be considered in sample size calculations for studies to replicate markers identified in exploratory analyses.

## Background

Using specimens, including serum, plasma or tumor tissue, from biobanks is attractive for biomarker studies, as samples are readily available. For example, archived patient tissue specimens from prospective clinical trials can be used for establishing the medical utility of prognostic or predictive biomarkers in oncology [1]. Convenience samples from clinical centers and hospitals may be of use in biomarker discovery studies. The National Cancer Institute maintains a website http://resresources.nci.nih.gov that lists human specimen resources available to researchers, including specimens and data from patients with HIV-related malignancies, a repository of thyroid cancer specimens and clinical data from patients affected by the Chernobyl accident, normal and cancerous human tissue from the Cooperative Human Tissue Network (CHTN) and blood samples to validate blood-based biomarkers for early diagnosis of lung cancer. However, freezing specimens over long periods of time can alter levels of some of their components [2] causing decreases or increases in marker concentrations [3-5]. Among other factors, storage temperature [6-8] and storage time [3,9,10] are known to impact frozen samples. Thus, even in carefully collected and stored samples time alone can alter marker levels.

Our work was motivated by a biomarker discovery study at the Medical University of Innsbruck that aims to identify biomarkers to predict breast cancer recurrence. In that study, among other investigations frozen serum samples from women diagnosed with breast cancer at the Medical University of Innsbruck Hospital between 1994 and 2010 will be used to identify candidate markers that predict breast cancer recurrence within five years of initial diagnosis. These markers will then be validated in prospectively collected specimens.

While the focus of discovery is the testing of association of markers with outcome, sample size considerations for validation studies are often based on estimated effect sizes seen in discovery studies. Any substantial bias in the effect sizes seen in the discovery effort will thus result in sample sizes of the follow up study that are too small (if associations are overestimated) or lead to the analysis of too many costly biospecimens (if estimates are too low). Additionally, degradation in markers could lead to missed associations, i.e. increased numbers of false negative findings, as effects may be attenuated.

We used simulations to systematically assess the impact of changes in marker levels due to storage time on estimates of association of marker levels with outcome in case-control studies. Our simulations are based on parameters obtained from data from a small laboratory experiment, designed to assess the impact of degradation on measurements of two serum markers. We study two set-ups for our simulations, one when single markers are analyzed, and one situation when multiple markers are used. While the choices of parameters depend on the specific setting, our results can help to assess the potential magnitude of a bias in and to interpret findings from studies that use biospecimens stored over long periods of time.

## Methods

### Markers

Cancer antigen 15-3 (CA 15-3) is a circulating tumor marker which has been evaluated for use as a predictive parameter in breast cancer patients indicating recurrence and therapy response. CA 15-3, the product of *MUC1 *gene, is aberrantly over expressed in many adenocarcinomas in an underglycosylated form and then shed into the circulation [11]. High concentrations of CA 15-3 are associated with a high tumor load and therefore with poor prognosis [12]. Thus, postoperative measurement of CA 15-3 is widely used for clinical surveillance in patients with no evidence of disease and to monitor therapy in patients with advanced disease. Cancer antigen 125 (CA125), another mucin glycoprotein, is encoded by the *MUC16 *gene. Up to 80% of epithelial ovarian cancers express CA125 that is cleaved from the surface of ovarian cancer cells and shed into blood providing a useful biomarker for monitoring ovarian cancer [13].

### Laboratory Methods

There are numerous reports on the impact of storage time on levels of individual components measured in serum in the literature [3,5,8,10,14,15]. We selected two well-known markers and measured their degradation over time. CA 15-3 and CA-125 were determined using a microparticle enzyme immunoassay and the Abbott IMx analyzer according to the manufacturers' instructions. Serum samples were collected at the Medical University of Innsbruck, Austria, between 1997 and 2001. Sample analysis was performed first at sample collection (1997 - 2001) and then again in September 2009, after storage at -30°C until 2004 and at -50°C thereafter. Eleven samples were analyzed for CA 15-3, and nine for CA125. Of the nine samples three had CA125 measurements below the detection limit of the assay. These samples were not used when computing mean and median differences.

Table 1 shows the values of the markers measured at the time of collection and the corresponding values for the same samples measured in September 2009.

**Table 1:** Marker Concentration Changes

Date of sample collection	Concentration measured		% change
	at sample collection	Sept 2009	
	CA 15-3		

Nov 1997	166	187	12.65
Oct 1998	29	33	13.79
Apr 1995	10	12	20.00
Feb 2001	21	19	-9.52
Apr 2001	23	24	4.35
Feb 1999	33	34	3.03
Sep 2000	26	33	26.92
Sep 2000	24	33	37.50
Sep 2000	15	17	13.33
Sep 2000	12	16	33.33
Nov 1999	884	986	11.54

	CA125		

Feb 1999	83	96	15.66
Feb 1999	< LOD^†^	< LOD	
Feb 1999	< LOD	< LOD	
Feb 1999	51	69	35.29
Feb 1999	< LOD	< LOD	
Sep 2000	77	73	-5.19
Sep 2000	33	32	-3.03
Sep 1998	106	105	-0.94
Oct 1998	1273	2026	59.15

^† ^LOD = limit of detection

Concentrations of two markers, CA 15-3 and CA125, measured at the time of freezing and then again after a long term storage. Measurements with concentrations below the limit of detection were excluded from further analysis.

### Statistical Model

#### Single Marker Model

Let *Y_i _*be one if individual *i *experiences the outcome of interest, i.e. is a case, and zero otherwise and let *X_i _*be the values of a continuous marker for person *i*. We assume that in the source population that gives rise to our samples, the probability of outcome is given by the logistic regression model(1)

The key parameter of interest is the log-odds ratio *β *that measures the increase in risk for a unit increase in marker levels.

We assume that the biomarkers are measured in retrospectively obtained case-control samples, as this is practically the most relevant setting. That is, first *n *individuals with the outcome of interest ("cases")and n individuals without that outcome ("controls") are sampled based on their outcome status, and then their corresponding marker values *X *are obtained. In our motivating example cases are women who experience a breast cancer recurrence within five years of initial breast cancer diagnosis, and controls are breast cancer patients without a recurrence in that time period.

#### Storage Effects on Marker Measurements

Instead of the true marker measurement *X*, we observe the value *Z_t _*of the marker after the sample has been frozen for *t *time units, e.g. months or years. We assume that *Z_t _*relates to *X *through the linear relationship(2)

The additive noise is assumed to arise from a normal distribution . Without loss of generality we focus on discrete time points, *t *= 0, 1, 2, ..., *t_max _*= 10 in our simulations. In the laboratory experiments, the marker levels for CA 15-3 increased by about 15% over a period of 10 years (Table 1). Because no intermediate measurements are available from our small laboratory study, the true pattern of change over time is unknown. Thus, we used three different sets of coefficients *b_j,t _*with *j *= 1, 2, 3, reflecting linear, exponential and logarithmic changes for the marker levels over time. Each set of coefficients was chosen to result in an increase of 15% after ten years of storage.

For the linear function, , the yearly increase in marker levels was set to 1.5%. To model the non-linear increases in marker levels, we estimated coefficients  and  based on an approximated Fibonacci series *f_t_*, where *f*_0 _= 0, *f*_1 _= 1, *f*_2 _= 2, and *f_t _*= *f*_*t*-1 _+ *f*_*t*-2 _for *t *= 2, ..., 10. For the exponential function  we normalized *f_t _*so that  was 15%.(3)

For a logarithmic increase we used coefficients(4)

**Figure 1:**
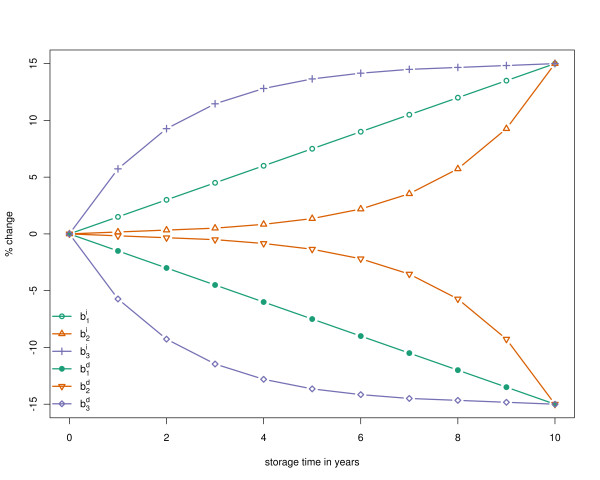
**Choices of *b_t_***. Three functions  model an increase in marker levels of 15% at *t *= 10, and three function  model a decrease of 15% at *t *= 10.

To simulate decreases in marker values over time, we used . All of these functions are plotted in Figure 1.

It is also possible to analytically assess the bias in estimates of in (1) when *Z_t _*is used instead of the true marker value *X *to estimate the association with disease. From (2) we get that *X *conditional on the measured *Z_t _*has a normal distribution, , where . Then using results from Carroll et al. [16]:(5)

Where . For multiple, correlated markers, which we study in the next section, a closed form analytical expression equivalent to (5) is not readily available.

#### Multiple Markers Model

We also studied a practically more relevant setting, namely that multiple markers are assessed in relation to outcome. We generated samples of *p *= 10 markers *X *= (*X*_1_, ..., *X_p_*) from a multivariate normal distribution, *X *~ *MVN*(0,Ω). We studied two choices of covariance structure: first, we let Ω = (*ω_ij_*) be the identity matrix, and second we assumed that the markers were equally correlated, with *corr*(*X_i_*, *X_j_*) = *ρ*, *i *≠ *j *for various choices of *ρ*.

We first assumed that only one marker, *X*_1_, was truly associated with outcome *Y*, and simulated *Y *from the model(6)

We also then let three of the markers, *X*_1_, *X*_2 _and *X*_3_, be associated with the outcome,(7)

In the simulations we let each marker change over time based on equation (2) independently of the other markers for *t *= 0, 1, 2, ..., *t_max _*= 10. For *X*_1 _the change over ten years was 15%, and for each of the other markers we randomly selected a coefficient *b_it _*from a uniform distribution on the interval [-0.2, 0.2] and used the chosen *b_it _*in equation (2). We thus allowed only increases or decreases of 20% or less over ten years.

### Simulations

To obtain case-control samples, we first prospectively generated a cohort of markers and outcome values (*Y_i_*, *X_i_*), *i *= 1, ..., *N*. We drew *X_i _*from a normal distribution, *X *~ *N*(0, 1), and then generated *Y_i _*given *X_i _*from a binomial distribution with *P*(*Y_i _*= 1|*X_i_*) given in equation (1) for *i *= 1, ..., *N*. We then randomly sampled *n *cases and *n *controls from the cohort to create our case-control sample.

For the single marker setting, we then fit a logistic regression model with *Z_t _*instead of *X *to the case-control data,(8)

and obtained the maximum likelihood estimate (MLE)  that characterizes the association of outcome with the marker measured after time *t *in storage.

For each setting of the parameters and for each choice of *b_t _*in (2), we simulated 1000 datasets for each sample size, *n *= 75 and *n *= 200 cases and the same number of controls for the single marker simulations, and *n *= 250 and *n *= 500 for the multiple marker settings. We also fit a logistic regression model based on the marker level *X *at time *t *= 0 that corresponds to no time related change in marker levels.

For the multiple marker setting, we analyzed the data using two different models. First, we fit separate logistic regression models for each marker,(9)

We also estimated regression coefficients for every time step from a joint model,(10)

In addition to the bias, we also assessed the power to identify true associations. When we fit separate models (9), we used a Bonferroni corrected type 1 error level *α *= 0.05/*p *to account for multiple testing. For the setting (10) we tested the null hypothesis  using a chi-square test with *p *degrees of freedom. Letting  be the vector of parameter estimates of the coefficients in (10), and  denote the corresponding estimated covariance matrix, we computed(11)

Of course model (10) can only be fit to data when *p *is substantially smaller than the available sample size, while model (9) does not have this limitation. For the multivariate simulations we computed the power, that is the number of times the null hypothesis is rejected over all simulations.

## Results

### Laboratory Experiment

On average both CA 15-3 and CA125 levels increased with increasing time in storage, CA 15-3 levels increased by 15.18% (*standard error *4.14) and CA125 16.82% (*standard error *10.533) over approximately ten years (Table 1). This increase is most likely due to evaporation of sample material attributed to the usage of sample tubes with tops that did not seal as well as the newer ones. A similar evaporating effect was reported by Burtis et al. [17]. Alternatively, the standard used for the calibration of the assay may have decreased over the years, resulting in higher levels for the more recent analysis.

### Simulation Results

#### Single Marker Results

We simulated storage effects for a period of ten years for three functions () that resulted in a 15% increase of marker levels after *t *= 10 years, and three functions, (), that resulted in 15% decrease after *t *= 10 years. We let *μ *= -3 and *β *= 0.3 in model (1) that describes the relationship between the true marker levels and outcome. The error variance in model (2) for the change of the marker over time was  = 0.01. We analyzed the simulated data at three time points, at sample collection (*t *= 0), and after *t *= 5 and *t *= 10 years.

Table 2 shows the results for functions , that result in increases of marker levels and , that cause decreases of marker levels. The results in Table 2 are means over 1, 000 repetitions for each choice of sample size. Table 2 also shows the relative bias, computed as . As expected, the true association parameter *β *= 0.3 in (1) was estimated without bias for *t *= 0 for all sample sizes. For *t *= 5, the relative bias ranged from 2% for  to -9% for  for *n *= 75 cases and controls, and from 1% for  to -10% for  for *n *= 200 cases and controls. The small positive bias for *t *= 5 for  was not seen when the simulation was repeated with a different seed. The differences in relative bias reflect the differences in the shape of increase of marker values. As all functions were chosen to cause a 15% increase in marker levels after *t *= 10 years, all functions resulted in the same relative bias at *t *= 10, which ranged from -10% for *n *= 75 cases and controls to -11% for *n *= 200 cases and controls. For example, at *t *= 10 instead of *β *= 0.3 we obtained  = 0.269 for *n *= 75 cases and controls and  = 0.268 for *n *= 200 cases and controls, respectively. The findings for decaying markers levels were similar. Again, no bias was detected in the estimates for *t *= 0, while the relative bias ranged from 4% for  to 18% for  for *n *= 200 cases and controls. After *t *= 10 years in storage, the relative bias was around 20% for *n *= 75 and *n *= 200 cases and controls. These results agree well with what we computed from the analytical formula (5). For all settings we studied the model based standard error estimates were similar to the empirical standard error estimates and were thus not shown.

**Table 2:** Univariate Marker Results

	*n *= 75						*n *= 200					
	**increase over time**			**decrease over time**			**increase over time**			**decrase over time**		

t = 0												

												

	0.309	0.309	0.309	0.309	0.308	0.308	0.308	0.308	0.307	0.307	0.308	0.308

se.emp	0.005	0.005	0.005	0.005	0.005	0.005	0.003	0.003	0.003	0.003	0.003	0.003

rel.bias	0.029	0.029	0.029	0.03	0.028	0.028	0.026	0.026	0.024	0.024	0.026	0.026

rel.bias.sd	0.566	0.566	0.568	0.571	0.568	0.563	0.343	0.342	0.343	0.341	0.342	0.34

t = 5												

												

	0.288	0.305	0.272	0.334	0.312	0.356	0.287	0.304	0.271	0.331	0.312	0.355

se.emp	0.005	0.005	0.005	0.006	0.005	0.006	0.003	0.003	0.003	0.003	0.003	0.004

rel.bias	-0.041	0.015	-0.092	0.112	0.042	0.186	-0.044	0.013	-0.096	0.105	0.039	0.184

rel.bias.sd	0.527	0.559	0.5	0.617	0.576	0.65	0.319	0.337	0.302	0.368	0.346	0.393

t = 10												

												

	0.269	0.269	0.269	0.362	0.361	0.361	0.268	0.268	0.268	0.36	0.361	0.361

se.emp	0.005	0.005	0.005	0.006	0.006	0.006	0.003	0.003	0.003	0.004	0.004	0.004

rel.bias	-0.103	-0.103	-0.103	0.208	0.204	0.204	-0.106	-0.106	-0.107	0.199	0.202	0.202

rel.bias.sd	0.493	0.493	0.495	0.671	0.667	0.66	0.298	0.297	0.298	0.4	0.401	0.399

Mean values of the maximum likelihood estimates  of *β *= 0.3 after *t *= 0, 5, and 10 years for the various degradation functions, with empirical (*se.emp*) standard error and the relative bias . Simulations were performed with *μ *= -3, and sample sizes *n *= 75 and *n *= 200. Function *b*_1 _corresponds to a linear change, *b*_2 _exponential change and *b*_3 _logarithmic change in marker levels over time.

Results were similar for *β *= 0.5, *β *= 1.0, and *β *= -0.3, given in Additional File 1.

#### Multiple Marker Results

Table 3 presents results for the multiple marker simulations, when one marker was truly associated with outcome, but the model that was fit to the data included all ten markers simultaneously (10). The results were very similar to the single marker simulations, with biases of about 10% after ten years. Correlations among markers did not affect the results. For example, the effect estimate after five years were  = 0.285 and 0.281 for *n *= 250 and *n *= 500 for uncorrelated markers, and  = 0.282 and 0.278 for *n *= 250 and *n *= 500 for fairly strong correlations of *ρ *= 0.5. The power to test for association using separate test with a Bonferroni adjusted *α*-level was adequate only for *n *= 500 cases and *n *= 500 controls.

**Table 3:** Multivariate Marker Results: A Single Marker is associated with Outcome

	*uncorrelated*		*correlated *(*ρ *= 0.5)	
	***n *= 250**	***n *= 500**	***n *= 250**	***n *= 500**

t = 0				

	0.305	0.302	0.303	0.298

se.emp	0.091	0.064	0.128	0.093

rel.bias	0.018	0.005	0.009	-0.005

rel.bias.sd	0.304	0.213	0.426	0.309

power^†^	0.522	0.92	0.541	0.908

t = 5				

	0.285	0.281	0.282	0.278

se.emp	0.085	0.059	0.119	0.086

rel.bias	-0.052	-0.064	-0.058	-0.072

rel.bias.sd	0.282	0.198	0.398	0.287

power	0.527	0.926	0.546	0.908

t = 10				

	0.266	0.263	0.264	0.261

se.emp	0.08	0.055	0.112	0.08

rel.bias	-0.114	-0.124	-0.121	-0.13

rel.bias.sd	0.266	0.185	0.372	0.268

power	0.532	0.929	0.55	0.91

Results for simulations based on a multivariate setting with 10 markers, where only *X*_1 _is associated with disease outcome with true *β *= 0.3, and *μ *= -3. Levels of *X*_1 _increases 1.5% per year. Simulations were performed with sample sizes *n *= 250 and *n *= 500. ^† ^The power is calculated as the number of rejected null hypotheses over all simulations.

Table 4 shows the results when three of the ten markers were associated with disease outcome. The true association parameters in equation (7) were *β*_1 _= 0.3, *β*_2 _= 0.2 and *β*_3 _= 0.2. The changes in marker levels after ten years were 15%, 20% and 10% for *X*_1_, *X*_2 _and *X*_3_, respectively. After *t *= 10 years the bias in the association estimate for marker *X*_1 _was similar to the single marker case, and the case when only one of ten markers was associated with outcome, with  = 0.261, with a 13% underestimate of true risk. For the other two markers the log odds ratio estimates after ten years were  = 0.169 and  = 0.182, corresponding to 15.5% and 9% relative bias. The power of a test for association using a ten degree of freedom chi-square test was above 90% even for a sample size of *n *= 250 cases and *n *= 250 controls.

**Table 4:** Multivariate Marker Results: Three Markers are associated with Outcome

	*X*1	*X*2	*X*3
true *β*	0.3	0.2	0.2

perc.change	0.150	0.20	0.10

*b^i^*	1	2	3

t = 0			

	0.3	0.202	0.2

se.emp	0.131	0.13	0.13

rel.bias	-0.001	0.012	0.002

rel.bias.sd	0.435	0.652	0.648

power^†^	0.996		

t = 5			

	0.279	0.199	0.184

se.emp	0.122	0.126	0.118

rel.bias	-0.068	-0.003	-0.078

rel.bias.sd	0.405	0.630	0.591

power	0.995		

t = 10			

	0.261	0.169	0.182

se.emp	0.113	0.108	0.117

rel.bias	-0.131	-0.155	-0.090

rel.bias.sd	0.376	0.538	0.584

power	0.995		

Results for simulations based on a multivariate setting 10 with correlated markers, with 250 cases and 250 controls, *μ *= -3, and *ρ *= 0.5. The first three markers *X*_1_, *X*_2_, and *X*_3 _are associated with outcome. ^† ^The power is calculated as the number of rejected null hypotheses over all simulations. Function *b*_1 _corresponds to a linear change, *b*_2 _exponential change and *b*_3 _logarithmic change in marker levels over time.

## Discussion

In this paper we quantified the impact of changes of marker concentrations in serum over time on estimates of association of marker levels with disease outcome in case-control studies. We studied several monotone functions (linear, exponential, logarithmic) of changes over time that captured increases as well as decreases in marker levels. All functions were designed so that after ten years the change in levels was a decrease or increase by 15%. This percent change was chosen based on observations from a small pilot study. Thus, for all different functions that were used to model markers changes the bias seen in the association parameter after ten years was the same, but for intermediate time points the magnitudes of biases differed, as the amount of change varied for different functions. For a 15% increase in marker levels, estimated log-odds ratios showed a relative bias of -10%, and for a 15% decrease in marker levels, log-odds ratios were overestimated, with a relative bias of about 20%. We assessed single markers as well as multiple correlated markers. The findings were similar, regardless of correlations.

While one could avoid this problem by using fresh samples, often, in prospective cohorts serum and blood are collected at baseline and at regular time intervals thereafter, and are used subsequently to assess markers for diagnosis or to estimate disease associations in nested case-control samples. This was the design that was used by investigators participating in the evaluation of biomarkers for early detection of ovarian cancer in the Prostate, Lung Ovarian and Colorectal (PLCO) cancer screening study.

If a biased estimate of true effect sizes due to systematic changes in biomarker levels is obtained in a discovery effort, this could lead to under- or overestimation of sample size for subsequent validation studies, and thus either compromise power to detect true effect sizes, or cause resources to be wasted. For example, for a case-controls study with one control per case to detect an odds ratio of 2.0 for a binary exposure that has prevalence 0.2 among controls with 80% power and a type one level of 5%, one needs a sample size of 172 cases and 172 controls. If the effect size is overestimated by 13%, leading to the biased odds ratio of 2.2, investigators may wrongly select 130 cases and 130 controls for the follow up study, causing the power to detect the true odds ratio of 2.0 to be 0.68.

The impact of storage effects on the loss of power to detect associations of multiple markers due to poor storage conditions was also assessed in [18], but no estimates of bias were presented in that study.

If the amount of degradation is known from previous experiments, one could attempt to correct the bias in the obtained estimates before designing follow up studies. For a small number of markers changes in concentrations over time have been reported [4,15,19]. However, such information is typically not available in discovery studies where one aims to identify novel markers. In addition, while many changes were monotonic in time [14], the number of freeze-thaw cycles [10,19,20] and changes in storage conditions can cause more drastic changes. This also happened at the Medical University of Innsbruck, where storage temperature changed from -30°C for samples stored until 2004 to -50°C for samples stored and collected after 2004.

For investigators interested in validating new markers prospectively, a small pilot study that measures levels of marker candidates identified in archived samples again in fresh samples to obtain estimates of changes in levels may help better plan a large scale effort.

We assumed that the degradation was non-differential by case-control status. However, it is conceivable that degradation in serum from cases is different than those in serum from controls. While it would be interesting to assess the impact of differential misclassification, it is difficult to obtain realistic choices for parameters that could be used in a simulation study.

In summary, our results provide investigators planning exploratory biomarker studies with data on biases due to changes in marker levels that may aid in interpreting findings and planning future validation studies.

## Conclusion

The increase or decrease in markers measured in stored specimens due to changes over time can bias estimates of association between biomarkers and disease outcomes. If such biased estimates are then used as the basis for sample size computations for subsequent validation studies, this can lead to low power due to overestimated effects or wasted resources, if true effect sizes are underestimated.

## Competing interests

The authors declare that they have no competing interests.

## Authors' contributions

RMP conceived the simulation studies, interpreted the data, and led the drafting and writing of the manuscript. HF conceived and executed the laboratory studies and took part in editing the manuscript. KGK, WOH, and LAJM performed the simulation studies and took part in writing the manuscript. AG initiated the study, contributed to the study design, and took part in editing the manuscript. All authors read and approved the final manuscript.

## Supplementary Material

Additional file 1**Univariate Marker Results for *β *= 0.5, *β *= 1, and *β *= -0.3**. Mean values of the maximum likelihood estimates  of *β *= 0.5, *β *= 1, and *β *= -0.3 after *t *= 0, 5, and 10 years for the various degradation functions, with empirical (*se.emp*) standard error and the relative bias of . Simulations were performed with *μ *= -3, and sample sizes *n *= 75 and *n *= 200. Function *b*_1 _corresponds to a linear change, *b*_2 _exponential change and *b*_3 _logarithmic change in marker levels over time.Click here for file
