# Supplementary material for: The impact of sample storage time on estimates of association in biomarker discovery studies
Source: J Clin Bioinforma. 2011 Mar 8;1:9. doi: 10.1186/2043-9113-1-9 (PMC3131186; doi:10.1186/2043-9113-1-9)
Supplement: Additional file 1 — Univariate Marker Results for β = 0.5, β = 1, and β = -0.3. Mean values of the maximum likelihood estimates of β = 0.5, β = 1, and β = -0.3 after t = 0, 5, and 10 years for the various degradation functions, with empirical (se.emp) standard error and the relative bias of . Simulations were performed with μ = -3, and sample sizes n = 75 and n = 200. Function b1 corresponds to a linear change, b2 exponential change and b3 logarithmic change in marker levels over time. [file 2043-9113-1-9-S1.PDF]

Supplementary Table 1 -
Univariate marker results for $\beta = 0.5$

Mean values of the maximum likelihood estimates $\hat{\beta}^*_t$ after $t = 0, 5$, and 10 years for the various degradation functions, and their empirical (*se.emp*) standard error and the relative bias of $\hat{\beta}^*_t$. Simulations were performed with $\mu = -3$, and sample sizes $n = 75$ and $n = 200$.

	$n = 75$						$n = 200$					
	increase over time			decrease over time			increase over time			decrease over time		
t=0												
	b^i_1	b^i_2	b^i_3	b^d_1	b^d_2	b^d_3	b^i_1	b^i_2	b^i_3	b^d_1	b^d_2	b^d_3
$\hat{\beta}^*_0$	0.511	0.51	0.511	0.51	0.51	0.51	0.509	0.509	0.509	0.509	0.509	0.51
se.emp	0.006	0.006	0.006	0.006	0.006	0.006	0.003	0.003	0.003	0.003	0.003	0.003
rel.bias	0.022	0.02	0.021	0.02	0.019	0.021	0.019	0.019	0.018	0.017	0.019	0.019
rel.bias.sd	0.355	0.356	0.357	0.354	0.355	0.358	0.215	0.214	0.214	0.216	0.214	0.215
t=5												
	b^i_1	b^i_2	b^i_3	b^d_1	b^d_2	b^d_3	b^i_1	b^i_2	b^i_3	b^d_1	b^d_2	b^d_3
$\hat{\beta}^*_5$	0.476	0.503	0.45	0.55	0.517	0.589	0.475	0.503	0.449	0.549	0.516	0.588
se.emp	0.005	0.006	0.005	0.006	0.006	0.007	0.003	0.003	0.003	0.004	0.003	0.004
rel.bias	-0.048	0.007	-0.099	0.101	0.033	0.178	-0.051	0.006	-0.102	0.098	0.032	0.176
rel.bias.sd	0.331	0.351	0.314	0.383	0.36	0.415	0.2	0.211	0.189	0.234	0.217	0.249
t=10												
	b^i_1	b^i_2	b^i_3	b^d_1	b^d_2	b^d_3	b^i_1	b^i_2	b^i_3	b^d_1	b^d_2	b^d_3
$\hat{\beta}^*_{10}$	0.445	0.445	0.445	0.598	0.597	0.598	0.444	0.444	0.444	0.596	0.597	0.597
se.emp	0.005	0.005	0.005	0.007	0.007	0.007	0.003	0.003	0.003	0.004	0.004	0.004
rel.bias	-0.109	-0.111	-0.11	0.195	0.195	0.196	-0.112	-0.112	-0.113	0.192	0.194	0.195
rel.bias.sd	0.31	0.31	0.311	0.416	0.417	0.421	0.187	0.187	0.187	0.254	0.251	0.252

Supplementary Table 2 -
Univariate marker results for $\beta = 1$

Mean values of the maximum likelihood estimates $\hat{\beta}^*_t$ after $t = 0, 5$, and 10 years for the various degradation functions, and their empirical (*se.emp*) standard error and the relative bias of $\hat{\beta}^*_t$. Simulations were performed with $\mu = -3$, and sample sizes $n = 75$ and $n = 200$.

	$n = 75$						$n = 200$					
	increase over time			decrease over time			increase over time			decrease over time		
t=0												
	b^i_1	b^i_2	b^i_3	b^d_1	b^d_2	b^d_3	b^i_1	b^i_2	b^i_3	b^d_1	b^d_2	b^d_3
$\hat{\beta}^*_0$	1.02	1.019	1.02	1.019	1.018	1.019	0.999	0.999	0.998	0.998	0.999	0.999
se.emp	0.007	0.007	0.007	0.007	0.007	0.007	0.004	0.004	0.004	0.004	0.004	0.004
rel.bias	0.02	0.019	0.02	0.019	0.018	0.019	-0.001	-0.001	-0.002	-0.002	-0.001	-0.001
rel.bias.sd	0.21	0.212	0.21	0.212	0.209	0.211	0.124	0.124	0.124	0.124	0.124	0.124
t=10												
	b^i_1	b^i_2	b^i_3	b^d_1	b^d_2	b^d_3	b^i_1	b^i_2	b^i_3	b^d_1	b^d_2	b^d_3
$\hat{\beta}^*_5$	0.95	1.005	0.899	1.1	1.031	1.176	0.931	0.986	0.881	1.077	1.013	1.153
se.emp	0.006	0.007	0.006	0.007	0.007	0.008	0.004	0.004	0.003	0.004	0.004	0.005
rel.bias	-0.05	0.005	-0.101	0.1	0.031	0.176	-0.069	-0.014	-0.119	0.077	0.013	0.153
rel.bias.sd	0.196	0.209	0.185	0.229	0.212	0.244	0.116	0.122	0.109	0.134	0.125	0.144
t=10												
	b^i_1	b^i_2	b^i_3	b^d_1	b^d_2	b^d_3	b^i_1	b^i_2	b^i_3	b^d_1	b^d_2	b^d_3
$\hat{\beta}^*_{10}$	0.889	0.888	0.889	1.195	1.192	1.195	0.871	0.871	0.871	1.17	1.171	1.171
se.emp	0.006	0.006	0.006	0.008	0.008	0.008	0.003	0.003	0.003	0.005	0.005	0.005
rel.bias	-0.111	-0.112	-0.111	0.195	0.192	0.195	-0.129	-0.129	-0.129	0.17	0.171	0.171
rel.bias.sd	0.183	0.184	0.183	0.249	0.246	0.247	0.108	0.108	0.108	0.145	0.145	0.146

Supplementary Table 3 - Univariate marker results for $\beta = -0.3$

Mean values of the maximum likelihood estimates $\hat{\beta}_t^*$ after $t = 0, 5$, and 10 years for the various degradation functions, and their empirical ($se.emp$) standard error and the relative bias of $\hat{\beta}_t^*$. Simulations were performed with $\mu = -3$, and sample sizes $n = 75$ and $n = 200$.

	$n = 75$						$n = 200$					
	increase over time			decrease over time			increase over time			decrease over time		
t=0												
	b_1^i	b_2^i	b_3^i	b_1^d	b_2^d	b_3^d	b_1^i	b_2^i	b_3^i	b_1^d	b_2^d	b_3^d
$\hat{\beta}_0^*$	-0.299	-0.299	-0.299	-0.299	-0.3	-0.299	-0.296	-0.296	-0.297	-0.297	-0.296	-0.296
se.emp	0.006	0.006	0.006	0.006	0.006	0.005	0.003	0.003	0.003	0.003	0.003	0.003
rel.bias	-0.003	-0.003	-0.004	-0.004	-0.001	-0.002	-0.013	-0.013	-0.011	-0.01	-0.012	-0.013
rel.bias.sd	0.582	0.582	0.584	0.581	0.582	0.578	0.342	0.343	0.342	0.344	0.343	0.345
t=5												
	b_1^i	b_2^i	b_3^i	b_1^d	b_2^d	b_3^d	b_1^i	b_2^i	b_3^i	b_1^d	b_2^d	b_3^d
$\hat{\beta}_5^*$	-0.279	-0.295	-0.264	-0.322	-0.304	-0.345	-0.276	-0.292	-0.262	-0.321	-0.3	-0.342
se.emp	0.005	0.005	0.005	0.006	0.006	0.006	0.003	0.003	0.003	0.004	0.003	0.004
rel.bias	-0.071	-0.016	-0.122	0.074	0.012	0.151	-0.08	-0.026	-0.128	0.068	0.001	0.139
rel.bias.sd	0.542	0.574	0.514	0.628	0.59	0.667	0.319	0.338	0.302	0.372	0.347	0.399
t=10												
	b_1^i	b_2^i	b_3^i	b_1^d	b_2^d	b_3^d	b_1^i	b_2^i	b_3^i	b_1^d	b_2^d	b_3^d
$\hat{\beta}_{10}^*$	-0.261	-0.261	-0.26	-0.35	-0.351	-0.351	-0.258	-0.258	-0.259	-0.348	-0.347	-0.347
se.emp	0.005	0.005	0.005	0.006	0.006	0.006	0.003	0.003	0.003	0.004	0.004	0.004
rel.bias	-0.131	-0.131	-0.132	0.166	0.171	0.169	-0.139	-0.139	-0.138	0.16	0.158	0.157
rel.bias.sd	0.507	0.507	0.508	0.682	0.683	0.678	0.298	0.299	0.298	0.404	0.402	0.405
